# The Role of Complete Blood Count-Derived Inflammatory Biomarkers as Predictors of Infection After Acute Ischemic Stroke: A Single-Center Retrospective Study

**DOI:** 10.3390/medicina60122076

**Published:** 2024-12-18

**Authors:** Weny Rinawati, Abdulloh Machin, Aryati Aryati

**Affiliations:** 1 Doctoral Program of Medical Science, Faculty of Medicine, Universitas Airlangga, Surabaya 60132, Indonesia; weny.rinawati-2022@fk.unair.ac.id; 2 Laboratory and Blood Bank, Department of Clinical Pathology, National Brain Center Hospital Prof. Dr. dr. Mahar Mardjono, Jakarta 13630, Indonesia; 3 Department of Neurology, Faculty of Medicine, Universitas Airlangga, Surabaya 60132, Indonesia; 4 Airlangga University Hospital, Surabaya 60115, Indonesia; 5 Dr. Soetomo General Academic Hospital, Surabaya 60132, Indonesia; 6 Department of Clinical Pathology, Faculty of Medicine, Universitas Airlangga, Surabaya 60132, Indonesia

**Keywords:** blood count, biomarker, infection, inflammation, ischemic stroke, lymphocyte, monocyte, neutrophil, platelet, post-stroke infection

## Abstract

*Background and Objectives*: Although a wide range of hematological parameters are used as blood-based inflammatory biomarkers, the role of complete blood count-derived inflammatory biomarkers in infection after acute ischemic stroke (AIS) is modest. Therefore, this study aimed to explore complete blood count-derived inflammatory biomarkers as predictors of infection after AIS. *Materials and Methods*: A single-center retrospective cross-sectional study was carried out at the National Brain Center Hospital Prof. Dr. dr. Mahar Mardjono, Jakarta, Indonesia, between 1 October 2023, and 31 March 2024, using medical records of hospitalized first-ever ischemic stroke patients who underwent a complete blood count within 24 h of admission. Based on complete blood count-derived inflammatory biomarkers, this study included absolute numbers and related ratios or indices. *Results*: In total, 163 patients met the study criteria. The diagnosis of infection after AIS was established using reliable clinical symptoms and/or guidelines of the disease. According to the status of infection after AIS, the subjects were categorized into two groups, including 24 patients in the infection group and 139 patients in the non-infection group. Biomarkers that had significant accuracy (higher sensitivity and specificity, respectively) in predicting infection were the leukocyte count (LC; 70.8%, 74.1%, *p* < 0.001), absolute neutrophil count (ANC; 66.7%, 79.9%, *p* < 0.001), absolute monocyte count (AMC; 75.0%, 63.3%, *p* = 0.001), neutrophil to lymphocyte ratio (NLR; 62.5%, 71.9%, *p* = 0.003), derivative NLR (dNLR; 50.0%, 78.4%, *p* = 0.003), monocyte–granulocyte to lymphocyte ratio (MGLR; 62.5%, 73.0%, *p* = 0.003), systemic inflammatory response index (SIRI; 62.5%, 79.0%, *p* = 0.001), and systemic immune inflammation index (SII; 87.5%, 44.0%, *p* = 0.012) with chances of 74.4%, 75.4%, 71.0%, 69.0%, 68.7%, 69.3%, 73.4%, and 66.2%, respectively. *Conclusions*: Considering the overall ROC curve used to evaluate the complete blood count-derived inflammatory biomarkers, ANC has a better ability to predict infection in AIS patients, as denoted by the highest AUC, suggesting a 75.4% chance of correctly discriminating patients with infection after stroke.

## 1. Introduction

There has been a significant increase in the incidence of stroke, with the incidence doubling in the last four centuries, especially in developing countries with moderate or low per capita income [1]. The outcome following a stroke is highly unpredictable and is influenced by variables specific to the patient, including age and the severity of the stroke, as well as preventable stroke-related complications, such as infections [2]. Prior meta-analyses revealed that the pooled total infection rate was 30%, with infections ranging from 5% to 65% [3]. Infection after stroke is a problem that needs to be solved since it has an impact on the high costs of stroke care and is linked to poor outcomes, prolonged length of stay, and increased risk of recurrent stroke [2,4,5].

Alternatives for preventing post-stroke infections that are being studied include administering preventive antibiotics. Providing this therapy produces varying outcomes. Several studies showed that administering preventive antibiotics to prevent infection does not reduce the overall incidence of infection after stroke, deliver better outcomes, or reduce mortality [3,5,6,7]. As greater antibiotic use can increase resistance rates, one main disadvantage of preventative antibacterial therapy is its potential to promote antibiotic resistance in common bacteria [2]. Antibiotics used as preventive treatment are believed to be ineffective if not administered to the right patient.

The first crucial step in the management of infection is an accurate and promptly determined diagnosis. Currently, biomarkers are among the primary methods for diagnosing infection and are frequently employed in the clinical setting, as they serve as rapid, unbiased, and accessible diagnostic tools for infection [8]. Biomarkers may help reduce the possibility of rising antibiotic resistance rates by assisting in the patient selection process for antibiotic therapy. Furthermore, early after a stroke, they may offer information on a patient’s prognosis, which may be crucial in guiding treatment choices. It may be preferable to treat problems early in a patient whose prognosis is expected to be positive to maximize the likelihood of improvement [2].

Nowadays, a wide range of hematological parameters are used as biomarkers. Compared to the others, the complete blood count (CBC) is a more user-friendly and less expensive method that is easily available in emergency settings, making it a possible biomarker of diseases [9]. In addition, inflammatory indexes that influence systemic inflammatory responses can be discovered by hematological parameters.

Several studies have suggested that the neutrophil to lymphocyte ratio (NLR) is a prognostic factor for a number of malignancies, heart conditions, and infections. The platelet to lymphocyte ratio (PLR) is a useful inflammatory biomarker that may also be used to predict a patient’s mortality and the prognosis of various illnesses. The lymphocyte to monocyte ratio (LMR) is inversely correlated with disease severity [10].

There were prior studies on blood count-related biomarkers in stroke. The neutrophil count (NC) had prognostic significance with cerebral small vessel disease [11]. The NLR can predict the neurological outcomes in patients with acute ischemic stroke with post-thrombolysis as well as LMR and PLR [12]. In addition, NLR and PLR have been documented to predict stroke prognosis even though their predictive power is limited [13]. Recent research has revealed that two novel inflammatory indices, the systemic immune inflammation index (SII) and systemic inflammation response index (SIRI), which comprise platelets and three subtypes of leukocytes, have a higher predictive value for prognosis [14].

Biomarkers have become commonplace in acute stroke management. To determine and choose the patients with acute ischemic stroke (AIS) who are most likely to benefit from reperfusion therapy, NLR may be a significant biomarker. The predictive value of NLR can be utilized to identify and track high-risk patients to inform early treatment and improve outcomes, but doing so requires the application of appropriate clinical decision-making tools and models [15].

Regarding the relationship with infection, previous studies highlighted that the NLR, systemic inflammation response index (SIRI), and systemic immune-inflammation index (SII) had roles as predictors of the occurrence and severity of pneumonia in patients with intracerebral hemorrhage [16]. In addition, SIRI was strongly correlated with the occurrence of sepsis, which is associated with all-cause mortality in stroke patients [13].

However, the role of complete blood count-derived inflammatory biomarkers in infection after AIS is modest. A neuroinflammatory cascade involving both cellular and innate inflammation is present in ischemic stroke. Therefore, this study aimed to explore complete blood count-derived inflammatory biomarkers as predictors of infection after AIS. Regarding the use of biomarkers, this study included absolute numbers and related ratios or indices.

## 2. Materials and Methods

### 2.1. Study Design and Participants

A single center carried out this retrospective cross-sectional study at a public tertiary teaching and referral neurology hospital that provides a variety of neurological health services, the National Brain Center Hospital (NBC) Prof. Dr. dr. Mahar Mardjono, Jakarta, Indonesia. Using consecutive sampling, we reviewed and collected secondary data from medical records of hospitalized ischemic stroke patients during the six months between 1 October 2023, and 31 March 2024. These were the inclusion criteria used while enrolling patients who were diagnosed with first-ever AIS, aged at least 18 years old, and underwent CBC within 24 h of admission. Since this study aimed to explore complete blood count-derived inflammatory biomarkers that were able to be obtained from CBC data from patients with infection after AIS, those who had an ischemic stroke more than seven days after the stroke onset, history of prior infection, hematological malignancy, and surgery were excluded from this study to remove potential misleading conditions that might influence the results [17].

Anonymous data were gathered from medical records. Data regarding demographics such as age and sex, clinical manifestation of infections, comorbidities, and CBC examination results were obtained from the medical records.

### 2.2. Definitions

The diagnosis of AIS was defined by reliable clinical symptoms and/or evidence of infarction from radiological examination (magnetic resonance imaging (MRI) or computed tomography scan (CT) [18,19], pursuant to a standard of clinical practice. The first-ever stroke was built upon a stroke event that had never occurred before. The patients were examined for infection within seven days after stroke onset [18]. Diagnosis of infection after AIS was established by reliable clinical symptoms and/or guidelines of the disease, for instance, pneumonia [20] or urinary tract infection (UTI) [20], in accordance with the guideline from the Centers for Disease Control (CDC); however, sepsis was defined using the Sepsis-3 criteria [21] described in previous studies [2,18,22,23]. According to the status of infection after AIS, the subjects were categorized into two groups, which were the infection and non-infection groups.

The cardiovascular risk factors that were included were hypertension, diabetes mellitus, hyperlipidemia, and hyperuricemia. Hypertension was identified as a history of using antihypertension, a systolic blood pressure ≥ 140 mm Hg, or diastolic blood pressure ≥ 90 mm Hg. Diabetes mellitus (DM) type 2 was determined by clinical diagnosis, current use of antihyperglycemic drugs, or a fasting glucose level > 126 mg/dL. Hyperlipidemia was determined based on a history of hyperlipidemia or the use of antihyperlipidemic agents, low-density lipoprotein (LDL) ≥ 160 mg/dL, or total cholesterol ≥ 240 mg/dL [24]. Hyperuricemia was indicated by uric acid ≥ 5.3 mg/dL. Serum fasting glucose, LDL, total cholesterol, and uric acid levels were measured using the chemiluminescent sandwich test principle (GLUC3, LDLC3, CHOL2, and UA2; Cobas^®^, Roche Diagnostics GmBH, Mannheim, Germany) with the clinical chemistry analyzer Cobas^®^ c501 (Roche Diagnostics GmBH, Mannheim, Germany) [25,26,27,28].

A complete blood count was conducted using an ethylenediaminetetraacetic acid (K_2_EDTA) blood specimen run in the Sysmex XN-1000 automated hematological analyzer (Sysmex Corporation, Kakogawa, Hyogo, Japan). The adult leukocyte count (LC) reference range was 5–10 × 10^9^/L. Moreover, the reference ranges for the leukocyte differential count, including absolute basophil count (ABC), absolute eosinophil count (AEC), absolute neutrophil count (ANC), absolute lymphocyte count (ALC), and absolute monocyte count (AMC), was 0–0.1 × 10^9^/L, 0.05–0.3 × 10^9^/L, 2.6–7.6 × 10^9^/L, 1–4 × 10^9^/L, and 0.1–0.8 × 10^9^/L, respectively. The adult platelet count (PC) reference range was 150–400 × 10^9^/L. Cellpenia refers to a cell count less than the lower limit reference range; on the contrary, cellcytosis or cellphilia refers to a cell count more than the upper limit reference range. Therefore, leukopenia and leukocytosis were defined as less than 5 × 10^9^/L and more than 10 × 10^9^/L, respectively, and thrombocytopenia and thrombocytosis were defined as less than 150 × 10^9^/L and more than 400 × 10^9^/L, respectively [29].

The ratio of each leukocyte subtype (basophil, eosinophil, neutrophil, or monocyte) to lymphocyte was based on the absolute count of each leukocyte subtype (ABC, AEC, ANC, or AMC) divided by the ALC. These ratios include the basophil to lymphocyte ratio (BLR = ABC/ALC), eosinophil to lymphocyte ratio (ELR = AEC/ALC), neutrophil to lymphocyte ratio (NLR = ANC/ALC), and monocyte to lymphocyte ratio (MLR = AMC/ALC). The derivative NLR (dNLR) was calculated by dividing the ANC with the leukocyte count (LC) minus the ANC (dNLR = ANC/{LC – ANC}). Meanwhile, the calculation of the ratio of monocyte–granulocyte to lymphocyte (MGLR) involved dividing the ALC by the LC minus lymphocytes (MGLR = {LC – ALC}/ALC).

The ratio of each leukocyte subtype (basophil, eosinophil, neutrophil, or lymphocytes) to monocyte was built upon the absolute count of each leukocyte subtype (ABC, AEC, ANC, or ALC) divided by the AMC, respectively. These ratios include the basophil to monocyte ratio (BMR = ABC/AMC), eosinophil to monocyte ratio (EMR = AEC/AMC), neutrophil to monocyte ratio (NMR = ANC/AMC), and lymphocyte to monocyte ratio (LMR = ALC/AMC).

The ratio of each leukocyte subtype (basophil, eosinophil, neutrophil, lymphocytes, or monocyte) to platelet was found by dividing the absolute count of each leukocyte subtype (ABC, AEC, ANC, ALC, or AMC) with the platelet count (PC), respectively. These ratios include the basophil to platelet ratio (BPR = ABC/PC), eosinophil to platelet ratio (EPR = AEC/PC), neutrophil to platelet ratio (NPR = ANC/PC), lymphocyte to platelet ratio (LPR = ALC/PC), and monocyte to platelet ratio (MPR = AMC/PC).

The platelet to neutrophil ratio (PNR) was defined as the PC divided by ANC (PNR = PC/ANC), whereas the platelet to lymphocyte ratio (PLR) was calculated as the PC divided by ALC (PLR = PC/ALC). The formula for the calculation of the systemic inflammatory response index (SIRI) involves dividing the product of the multiplication of ANC and AMC by ALC (SIRI = {ANC × AMC}/ALC), while the measure of systemic immune inflammation index (SII) was obtained by dividing the product of the multiplication of ANC and PC by ALC (SII = {ANC × PC}/ALC).

### 2.3. Data Analysis

This study utilized the Statistical Package for the Social Sciences v. 26 (IBM^®^ SPSS^®^ Statistics, Armonk, NY, USA) for analysis. Descriptive information about the distinct characteristics of the study participants was provided by considering the normality of the data distribution obtained using the Kolmogorov–Smirnov test. In descriptive statistics for continuous data, interquartile ranges (IQRs) and medians were used to present non-normally distributed data; at the same time, number (*n*) and percentage (%) were employed to show categorical variables. Characteristics differences between the two categorical variables of the infection and non-infection groups were compared using Chi-square analysis or Fisher’s exact test if the cell of the 2 × 2 table had an expected count of less than 5. Likewise, differences between two continuous variables were discovered using the Mann–Whitney U test. *p*-values were calculated from two-sided tests. Biomarkers’ cut-off points with the highest Youden index (Youden index = sensitivity + specificity – 1) were determined using the receiver operating characteristic (ROC) curves. The receiver operating characteristic (ROC) curve analysis was developed to assess the accuracy of inflammatory biomarkers obtained from complete blood counts in identifying AIS patients with or without infection. Each point on the ROC curve represented sensitivity and specificity. To determine the discriminating capability of complete blood count inflammatory biomarkers, the area under the ROC curve (AUC) was calculated. If the AUC was equal to 0.5 or more, the marker had some discrimination potential.

## 3. Results

**Figure 1:**
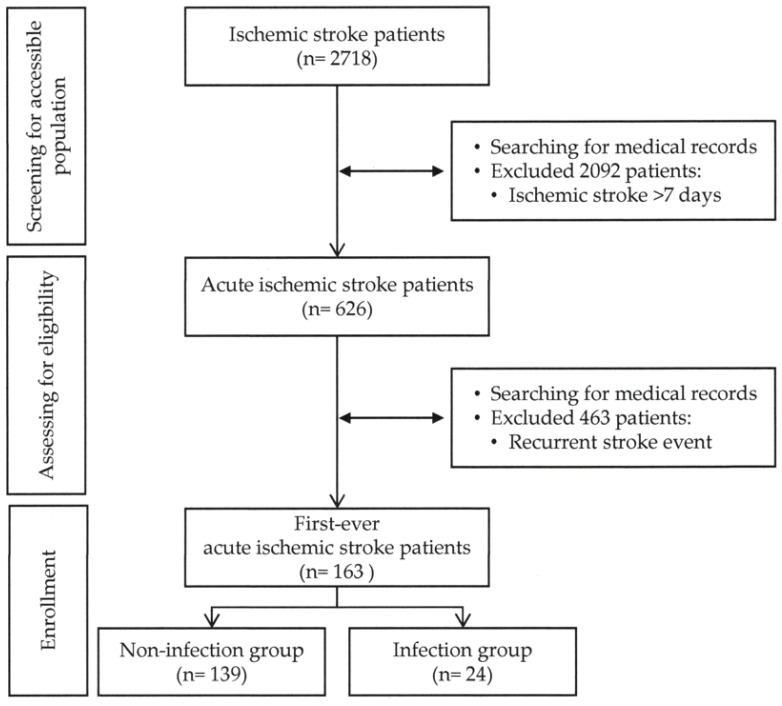
Flowchart of study participants.

There were 2718 patients who had an ischemic stroke between 1 October 2023, and 31 March 2024, based on the medical records of the hospitalized patients. Among those, 626 patients were diagnosed with acute ischemic stroke. After excluding 463 patients with a history of recurrent stroke, this study enrolled 163 participants, including 24 patients in the infection group (38.1%) and 139 patients in the non-infection group (61.9%) (Figure 1).

The study discovered that almost three-quarters of patients have hyperlipidemia followed by hypertension (59.5%) as the most common cardiovascular risk. Additionally, twelve patients developed infections within seven days, including seven pneumonia cases (4.3%), 4 UTIs (2.5%), and one sepsis case (0.6%). Despite the proportion being small, pneumonia was the most prevalent clinical manifestation of infection, after which came UTI. Most patients have normal platelet and leukocyte counts, including a normal leukocyte differential count. A summary of subject characteristics is listed in Table 1.

### 3.1. Characteristics of Infection and Non-Infection Groups

There were no differences in age groups, sex, and cardiovascular risk factors in the infection and non-infection groups of AIS patients. Moreover, there were no differences in the number of patients who had leukocytosis, normal leukocyte levels, leukopenia, thrombocytosis, normal thrombocyte levels, and thrombocytopenia in laboratory results.

**Figure 2:**
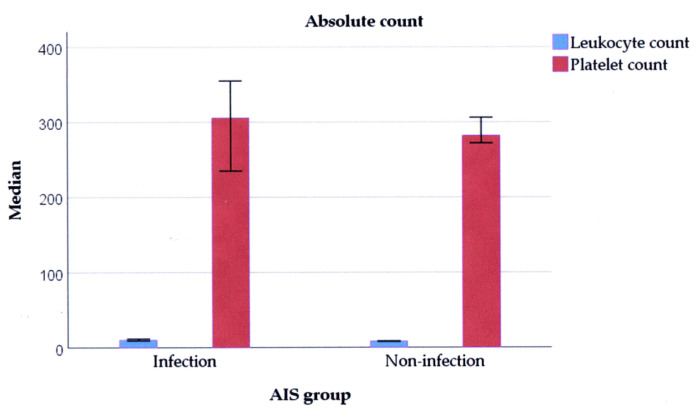
The median leukocyte count (*p* < 0.001) and platelet count (*p* = 0.592).

Regarding the leukocyte differential count, differences between neutrophils and lymphocytes were found between infection and non-infection patients. Regarding the leukocyte subtype, bivariate analysis indicated that the infection group had neutrophilia (37.5 vs. 12.9%, *p* = 0.016) and lymphocytopenia (50.0 vs. 22.3%, *p* = 0.007) compared to the non-infection group. On the other hand, weighing the cell count, the leukocyte count of the infection group was higher compared to that of the non-infection group (10.05 vs. 8.40 × 10^9^/L, *p* < 0.001), as well as ANC (7.70 vs. 5.47 × 10^9^/L, *p* < 0.001) and AMC (0.29 vs. 0.25 × 10^9^/L, *p* = 0.001) (Figure 2).

There were no differences in the ratios among each leukocyte subtype to lymphocytes, except the NLR (3.76 vs. 2.65, *p* = 0.003). Regarding the ratios of all granulocytes and monocytes, the ratio to lymphocytes (MGLR) of the infection group was also higher compared to the non-infection group (4.28 vs. 2.85, *p* = 0.003). In addition, the dNLR of the infection group was also higher than the non-infection group (2.71 vs. 1.94, *p* = 0.003). On the contrary, there were no differences in the ratios to monocytes between infection and non-infection groups. Regarding the platelet count, the ratio for each leukocyte subtype to platelets was not different, but the LPR of the infection group was lower compared to that of the non-infection group.

**Figure 3:**
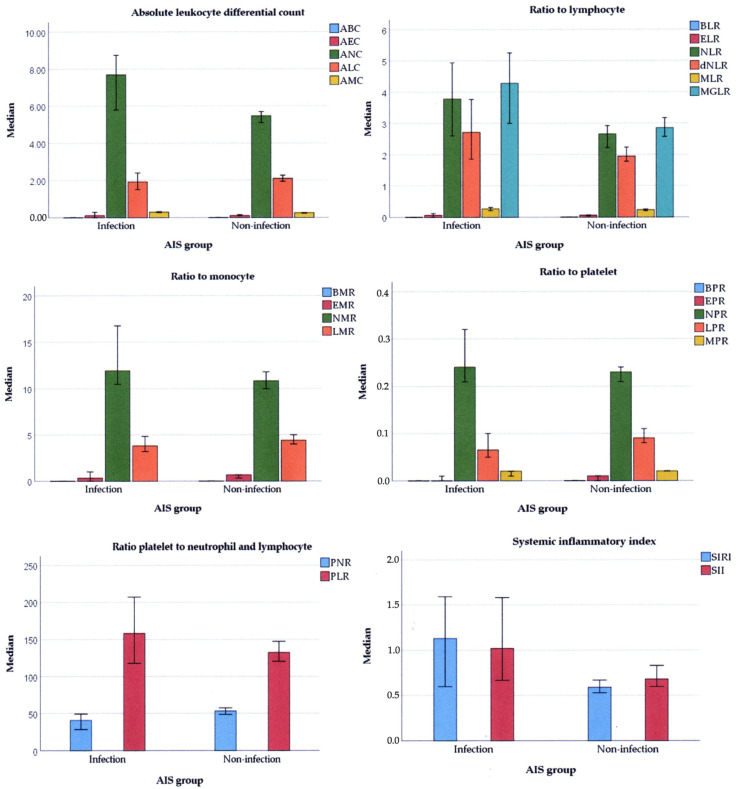
The medians of leukocyte and platelet indices between the infection and non-infection AIS groups. ABC: absolute basophil count, AEC: absolute eosinophil count, ANC: absolute neutrophil count, ALC: absolute lymphocyte count, AMC: absolute monocyte count, BLR: basophil to lymphocyte ratio, ELR: eosinophil to lymphocyte ratio, NLR: neutrophil to lymphocyte ratio, dNLR: derivative NLR, MLR: monocyte to lymphocyte ratio, MGLR: monocyte–granulocyte to lymphocyte ratio, BMR: basophil to monocyte ratio, EMR: eosinophil to monocyte ratio, NMR: neutrophil to monocyte ratio, LMR: lymphocyte to monocyte ratio, BPR: basophil to platelet ratio, MPR: monocyte to platelet ratio, PNR: platelet to neutrophil ratio, PLR: platelet to lymphocyte ratio, SIRI: systemic inflammatory response index, SII: systemic immune inflammation index.

The ratios of platelets to neutrophils and lymphocytes only demonstrated a difference in the PNR, which was lower in the infection group compared to the non-infection group (40.62 vs. 53.23, *p* = 0.001). Beyond that, there were differences in the systemic inflammatory index. The SIRI and SII of the infection group were higher compared to those of the non-infection group (1.13 vs. 0.59, *p* < 0.001; 1.02 vs. 0.68, *p* = 0.012, respectively) (Figure 3, Supplementary Table S1).

### 3.2. Performance of Complete Blood Count-Derived Inflammatory Biomarkers in Predicting Infection After AIS

**Figure 4:**
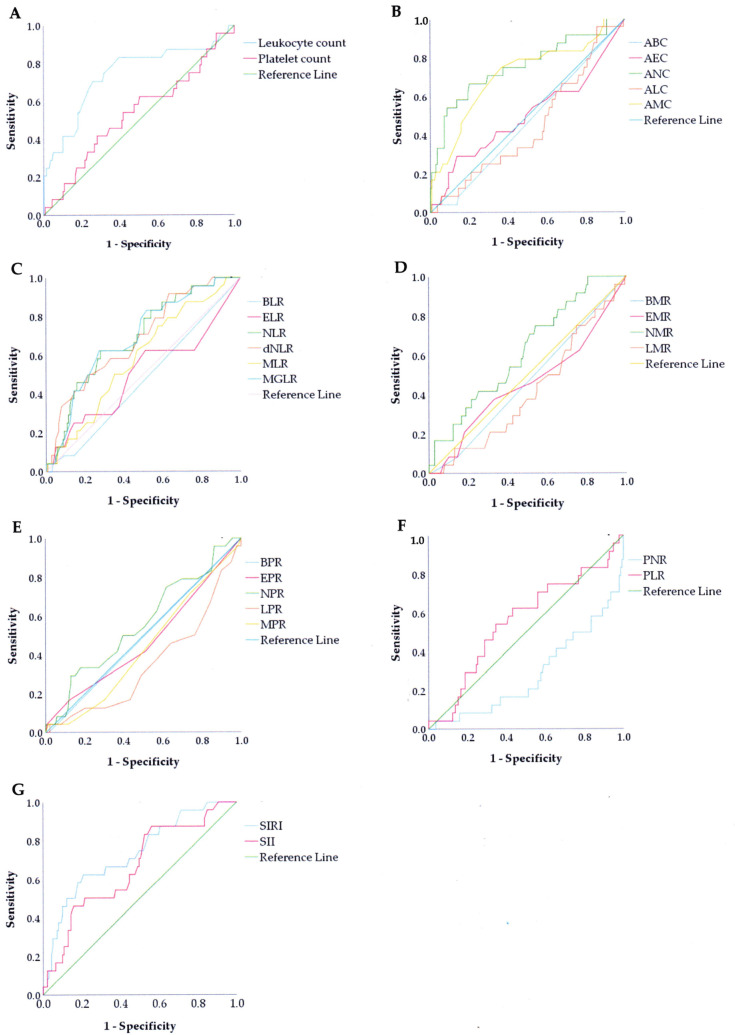
Receiver operating characteristic (ROC) curves of complete blood count-derived inflammatory biomarkers: (**A**) Leukocyte and platelet count, (**B**) Absolute leukocyte differential count, (**C**) Ratio to lymphocytes, (**D**) Ratio to monocytes, (**E**) Ratio to platelets, (**F**) Ratio of platelets to neutrophils and lymphocytes, and (**G**) Systemic inflammatory index.

Receiver operating characteristic (ROC) curves obtained for all of the blood-based inflammatory biomarkers showed that LC, ANC, AMC, NLR, dNLR, MGLR, LPR, PNR, SIRI, and SII had significant accuracy in predicting infection after AIS (Figure 4). Using the AUC to measure the discriminating power of different variables (with an AUC equal to 0.5 or more), biomarkers that were able to distinguish between the two groups were LC, ANC, AMC, NLR, dNLR, MGLR, SIRI, and SII with AUC values of 0.744, 0.754, 0.710, 0.690, 0.687, 0.734, and 0.662, respectively. The optimal cut-off was obtained from the Youden index (Supplementary Table S2).

## 4. Discussion

Although contrary to a population-based study by Corso et al. (2014), the findings for the first-ever AIS patients in this study were in line with the other studies [24], which included more males than females. The most common group was patients older than 60 years of age, and the patients’ median age was lower than that noted in most studies. Hyperlipidemia was the most prevalent comorbidity in this study, followed by DM. Based on the population-based study, patients who experienced their first-ever ischemic stroke at a relatively very old age were expected to be associated with additional comorbidities that impact long-term mortality after ischemic stroke, and the most common comorbidities that serve as vascular risk factors include hypertension followed by hypercholesterolemia [30].

Blood-based inflammatory biomarkers have recently been investigated for neurological pathologies. Regarding infection after AIS, several studies have already investigated the role of complete blood count-derived inflammatory biomarkers, yet those studies only inquired about extensively studied biomarkers, such as NLR, PLR, and LMR, as well as the novel inflammatory indices SIRI and SII. This study explored the hematological parameters and indices for most leukocytes, leukocyte subtypes, and platelet counts and indices that probably had better sensitivity and specificity to predict infection after AIS.

Following a stroke, there are notable alterations in the quantity and behavior of circulating immune cells. They become activated and secrete various cytokines and chemokines, which are critical in mediating the inflammatory response. This immune response is significant in the pathophysiology of stroke, as it can influence both recovery and overall outcomes. A well-regulated balance of these immune responses is important because excessive inflammation may lead to further neuronal damage, and appropriate modulation can promote tissue healing and recovery [17]. Besides infection, leukocytosis in ischemic stroke can also result from an ischemic event response in the absence of infection [31]. Despite the pathophysiological relationship among leukocyte count, infection, and AIS outcome, after adjusting for other risk factors that might be affected, complete blood count-derived inflammatory biomarkers exhibited independent predictive value for infection among AIS patients in a prior study [32]. Therefore, as stated in the exclusion criteria, this study eliminated any potential deceptive influences that could have an impact.

The primary function of leukocytes is to protect the host from pathogens by employing defense mechanisms called the innate (natural) and/or adaptive (acquired) immune systems [33]. Leukocytes are recruited, accumulate in tissues, and activate to destroy the microbes. Many of these reactions involve cytokines produced by dendritic cells, macrophages, and other types of cells during innate immune reactions [34]. In this study, the normal count of leukocytes dominated first-ever AIS patients with the leukocyte count median being similar to that reported in a previous study [17], but the leukocyte median of the infection group was higher compared to the non-infection group of AIS patients. The LC cut-off for distinguishing between the infection and non-infection groups of AIS patients was 8.60 × 10^9^/L, and its sensitivity and specificity were 66.7% and 79.9%, respectively. The sensitivity and specificity of this study were higher compared to prior studies [35,36]. A normal leukocyte count does not rule out the presence of disease, but leukocytosis or leukopenia is an essential clue to disease processes and deserves further investigation, including a leukocyte differential count to identify the concentration of the different types of leukocytes [33].

Platelets also have a significant role in inflammation and infection as the first cell recruited. The role is to use complex interactions among leukocyte and vascular endothelial cells, with coagulation and complement systems as mediators [37]. An inflammatory reaction can be exacerbated by alterations in microcirculation, augmented blood vessel permeability, platelet activation, and aggregation. Therefore, systemic inflammation is linked to thrombocytosis [38]. In this study, the normal count of platelets dominated first-ever AIS patients, with no difference in platelet medians between the groups of AIS patients. Though the PC median in this study was higher than that in previous studies [17,39], the PC cut-off cannot discriminate between the infection and non-infection groups of AIS patients. In addition, the ratio of each leukocyte subtype to platelets cannot be used.

During the acute phase of ischemic stroke in this study, which was the initial 7 days, pneumonia developed in most of the infection group patients, in accordance with previous studies. Pneumonia is the most recurrent infection that complicates acute ischemic stroke [22,23], as dysphagia is the most frequent complication following stroke [40]. Infection in an individual involves complex interactions between the host and the pathogen. Infection can be prevented by innate immunity by recognizing invading pathogen features and adaptive immunity through antigen-specific receptors. During infection, the innate immune system blocks the entry and eliminates or limits the growth of pathogens, and leukocyte recruitment as a cellular innate immune response causes inflammation initiation [34].

Nonetheless, an alteration in the leukocyte subtypes can cause an alteration in the leukocyte count [33]. Regarding the leukocyte subtype percentage, the infection group of AIS patients had neutrophilia and lymphocytopenia. However, based on absolute counts, the infection group of AIS patients had neutrophilia and monocytosis. The result of this study was consistent with that of another study [38]. The ANC and AMC in this study could distinguish between the infection and non-infection groups of AIS patients, with the ANC cut-off being 6.88 × 10^9^/L (sensitivity 66.7%, specificity 79.9%), which was higher compared to a prior study [37]. The AMC cut-off was 0.27 × 10^9^/L (sensitivity 75.0%, specificity 63.3%), which was lower compared to the previous study [36]. Neutrophils are the most numerous leukocyte subtype in peripheral blood. In the early innate response to the microbe, the major leukocyte subtypes recruited in inflammation are neutrophils and monocytes as the phagocytes. Phagocytes not only ingest microbes and dead cells but also destroy them in intracellular vesicles; thus, the innate immunity reactions effectively control and eradicate infection [34].

The innate immune system is interconnected with the adaptive immune system in many ways, with the innate immune response to microbes triggering adaptive immune responses and influencing the adaptive response nature. Humoral immunity and cell-mediated immunity are two types of adaptive immune responses that attempt to eliminate different types of microbes and are mediated by various immune system components. Some essential characteristics of all humoral and cell-mediated immune responses to foreign antigens are similar to those of the cells mediating responses. Antigen receptors expressed by lymphocytes vary and can be used for various foreign substances [34]. While inflammation results from demargination, redistribution, and increased apoptosis, lymphocytopenia may be a stress characteristic [39]. Increased apoptosis, redistribution, and margination of lymphocytes inside the lymphatic system are further mechanisms causing lymphocytopenia [38].

The cellular arm of the adaptive immune system influences eosinophil production and function. Eosinophils liberate substances that can neutralize basophil products, thereby downmodulating the allergic response [33]. In this study, there was no difference in percentages, counts, and indices involving eosinophils and basophils between the infection and non-infection groups of AIS patients. In another study, the eosinophil count was a predictor for a better outcome and lower mortality. Specifically, a decrease in the eosinophil count was associated with a worse outcome. Eosinophils are a source of tissue factors, provide a pro-coagulant phospholipid surface, and stimulate platelet activation with eosinophil granule contents [17].

Along with neutrophilia, lymphocytopenia, and monocytosis, the NLR, dNLR, and MGLR of the infection group were higher than the non-infection group of AIS patients, but the MLR was not statistically significant. The median value of the MLR in this study was lower than that in other studies exploring healthy subjects [41,42]. In the other study, MLR was used to identify the risk of influenza, malaria, and tuberculosis because it had larger probabilities for bacterial infection and low probabilities for viral infection [43].

The NLR was first proposed as a straightforward measure of stress and systemic inflammatory response syndrome (SIRS) in critically ill patients to assess the degree of sepsis and systemic infection, including bacteriemia [39]. It is a composite parameter that combines data from the innate immune response played by neutrophils and the adaptive regulatory arm of immunity played by lymphocytes, which indicates the balance between innate and adaptive immune responses. It represents a valuable biomarker of inflammation and stress together [17,39]. The NLR is a sensitive marker of acute, subacute, and/or chronic inflammation in association with infectious, non-infectious, and diseases with mixed etiology since it increases steadily as the disease progresses [32,39]. The NLR cut-off value to distinguish between the infection and non-infection groups of AIS patients in this study was 3.33 (sensitivity 62.5%, specificity 71.9%). Notwithstanding, this cut-off is lower compared to the prior study [32], but the median value was higher compared to the normal value in healthy adults [39,41,42] and corresponded to the cut-off value for bacterial infection in most studies of different races in the world (NLR ≥ 3–7) [39].

The dNLR cut-off value to discriminate between the infection and non-infection groups of AIS patients in this study was 0.28 (sensitivity 50.0%, specificity 78.4%), which is lower than that reported in a previous study [9]. The dNLR includes monocytes and other granulocytes by using the difference between the WBC and neutrophils in the denominator and is a potential new biomarker of systemic inflammation. Because of a pro-inflammatory environment, poorly differentiated and immature neutrophils can be released and rapidly increase neutrophil generation. Therefore, the dNLR will more comprehensively reflect this negative inflammation [9].

The MGLR was reported as a prognostic survival biomarker in several cancers that showed increasing frequency of recurrence [44]. In this study, the MGLR could discriminate between the infection and non-infection groups of AIS patients with a cut-off value of 3.66 (sensitivity 62.5%, specificity 73.0%), which was higher than that reported in another study [44].

SIRI and SII are novel biomarkers developed based on cells that have a unique relationship with one another and a role in immunology and inflammation. A rise in SIRI levels denotes a substantial pro-inflammatory response driven by neutrophils and monocytes, together with a diminished anti-inflammatory effect mediated by lymphocytes, whereas the potential use of SII originates from this relationship among neutrophils, lymphocytes, and platelets [38,45]. Although SIRI has been defined to be utilized as a prognostic marker in malignancy, its diagnostic performance has been examined in many diseases involving inflammation [38]. SIRI and SII incorporate three inflammatory cells, reflecting the immune and inflammatory system more accurately compared to other inflammatory indices that combine two cell types [45]. In this study, SIRI and SII had the ability to distinguish between the infection and non-infection groups of AIS patients with a cut-off value of 0.97 (sensitivity 62.5%, specificity 79.0%) and 0.61 (sensitivity 87.5%, specificity 44.0%), respectively.

However, in this study, considering an overall ROC curve to evaluate the complete blood count-derived inflammatory biomarkers, ANC has a better ability to predict AIS patients with and without infection, as denoted by the highest AUC, suggesting a 75.4% chance of correctly distinguishing these patients.

There are some drawbacks in this study. This result may need further validation if this system is utilized in a different setting since this study was undertaken at a single center. However, this hospital serves as a research area and is the tertiary center accommodating all reference cases in Indonesia. This study ruled out any potential misleading factors that could have an impact, but this does not eliminate the possibility that other factors influenced this study given the retrospective design. This study only explored a single measurement of the complete blood count-derived inflammatory biomarkers at admission to predict infection after the first-ever AIS. Consequently, future studies need to understand the dynamic of these biomarkers, inasmuch as inflammatory biomarkers may change.

## 5. Conclusions

Complete blood count-derived inflammatory biomarkers, including absolute numbers and related ratios or indices that influence systemic immune and inflammatory responses, exhibit the possibility to predict an infection after AIS. These biomarkers are promising because they are rapidly and easily obtained. Study findings showed that LC, ANC, AMC, NLR, dNLR, MGLR, LPR, PNR, SIRI, and SII had significant accuracy in predicting infection. Using the AUC as a measure of the discriminating power of different variables, biomarkers that correctly distinguished infection after the first-ever AIS were LC, ANC, AMC, NLR, dNLR, MGLR, SIRI, and SII with chances of 74.4%, 75.4%, 71.0%, 69.0%, 68.7%, 69.3%, 73.4%, and 66.2%, respectively. Using an overall ROC curve to evaluate the complete blood count-derived inflammatory biomarkers, ANC has a better ability to predict infection after AIS, as denoted by the highest AUC, suggesting a 75.4% chance of correctly discriminating patients.

## Figures and Tables

**Table 1:** Subject characteristics based on the medical records of 163 hospitalized patients, including 24 patients in the infection group and 139 patients in the non-infection group.

	Total		Infection		Non-Infection		*p*-Value
	*n*	%	*n*	%	*n*	%	
Subject	163	100	24	38.1	139	61.9	-
Age, median (IQR)	61.0 (17)		64.5 (21)		60.0 (16)		0.290
Age group, years, *n* (%)							
<60	73	44.8	7	29.2	66	47.5	0.096
≥60	90	55.2	17	70.8	73	52.5	
Sex, *n* (%)							
Male	96	58.9	12	50.0	84	60.4	0.337
Female	67	41.1	12	50.0	55	39.6	
Vascular risk factor, *n* (%)							
Hypertension	97	59.5	14	58.3	83	59.7	0.899
DM	68	41.7	12	50.0	56	40.3	0.373
Hyperlipidemia	118	72.4	20	83.3	98	70.5	0.194
Hyperuricemia	21	12.9	5	20.8	16	11.5	0.201
Clinical manifestation of infection, *n* (%)							
Pneumonia	7	4.3	7	29.2	-	-	<0.001 *
UTI	4	2.5	4	16.7	-	-	<0.001 *
Sepsis	1	0.6	1	4.2	-	-	0.147
Leukocyte, 10^9^/L, *n* (%)							
>10.00	37	22.7	12	50.0	25	18.0	1.000
5.00–10.00	125	76.7	12	50.0	113	81.3	-
<5.00	1	0.6	-	-	1	0.7	1.000
Leukocyte differential count, *n* (%)							
Basophil, *n* (%)							
>1	1	0.6	-	-	1	0.7	0.677
0–1	162	99.4	24	100.0	138	99.3	1.000
Eosinophil, *n* (%)							
>3	28	17.2	5	20.8	23	16.5	0.334
1–3	93	57.1	10	41.7	83	59.7	-
<1	42	25.8	9	37.5	33	23.7	0.099
Neutrophil, *n* (%)							
>76	27	16.6	9	37.5	18	12.9	0.016 *
52–76	126	77.3	15	62.5	111	79.9	-
<52	10	6.1	-	-	10	7.2	0.601
Lymphocyte, *n* (%)							
>40	6	3.7	-	-	6	4.3	1.000
20–40	114	69.9	12	50.0	102	73.4	-
<20	43	26.4	12	50.0	31	22.3	0.007 *
Monocyte, *n* (%)							
>8	15	9.2	1	4.2	14	10.1	0.698
2–8	145	89.0	22	91.7	123	88.5	-
<2	3	1.8	1	4.2	2	1.4	0.400
Platelet, 10^9^/L, *n* (%)							
>400.00	14	8.6	2	8.3	12	8.6	0.148
150.00–400.00	148	90.8	21	87.5	127	91.4	-
<150.00	1	0.6	1	4.2	-	-	1.000

* *p* < 0.05 indicates statistically significant related variables. IQR: interquartile range, DM: diabetes mellitus, UTI: urinary tract infection.

## Data Availability

The original contributions are presented in this study. Further inquiries can be directed to the corresponding author.

## Supplementary Materials

The following supporting information can be downloaded at: https://www.mdpi.com/article/10.3390/medicina60122076/s1, Table S1: Leukocyte and platelet count and indices of infection and non-infection groups; Table S2: ROC curve analysis of complete blood count-derived inflammatory biomarkers in infection after AIS.
