# Supplementary material for: The Role of Complete Blood Count-Derived Inflammatory Biomarkers as Predictors of Infection After Acute Ischemic Stroke: A Single-Center Retrospective Study
Source: Medicina (Kaunas). 2024 Dec 18;60(12):2076. doi: 10.3390/medicina60122076 (PMC11679367; doi:10.3390/medicina60122076)
Supplement: Supplementary file 1 [file medicina-60-02076-s001.zip › medicina-3329751-supplementary.pdf]

Supplementary Table S1. Leukocyte and platelet count and indices of the infection and non-infection groups.

	Total		Infection		Non-infection		*p*-value
	Median	IQR	Median	IQR	Median	IQR	
Leukocyte, 10^9/L	8.60	2.60	10.05	3.00	8.40	2.50	<0.001*
Absolute leukocyte differential count, 10^9/L							
ABC	0.00	0.00	0.00	0.00	0.00	0.00	0.423
AEC	0.11	0.22	0.11	0.29	0.11	0.17	1.000
ANC	5.61	2.42	7.70	3.63	5.47	2.28	<0.001*
ALC	2.06	1.11	1.92	1.12	2.10	1.10	0.419
AMC	0.26	0.08	0.29	0.07	0.25	0.09	0.001*
Ratio to lymphocyte							
BLR	0.00	0.00	0.00	0.00	0.00	0.00	0.456
ELR	0.06	0.10	0.06	0.15	0.06	0.07	0.960
NLR	2.72	2.15	3.76	2.43	2.65	1.76	0.003*
dNLR	2.03	1.41	2.71	2.08	1.94	1.13	0.003*
MLR	0.23	0.15	0.27	0.13	0.23	0.15	0.182
MGLR	3.00	2.33	4.28	2.56	2.85	1.87	0.003*
Ratio to monocyte							
BMR	0.00	0.00	0.00	0.00	0.00	0.00	0.450
EMR	0.67	1.00	0.33	1.00	0.67	0.67	0.375
NMR	11.00	6.20	11.92	9.09	10.83	5.97	0.058
LMR	4.33	3.02	3.79	2.06	4.40	3.15	0.198
Ratio to platelet							
BPR	0.00	0.00	0.00	0.00	0.00	0.00	0.556
EPR	0.00	0.01	0.00	0.01	0.01	0.01	0.631
NPR	0.23	0.09	0.24	0.13	0.23	0.09	0.338
LPR	0.09	0.06	0.07	0.05	0.09	0.05	0.020*
MPR	0.02	0.02	0.02	0.01	0.02	0.02	0.294
Platelet, 10^9/L	284.00	109.00	305.50	140.00	282.00	106.00	0.592
Ratio platelet to neutrophil and lymphocyte							
PNR	51.21	26.21	40.62	23.04	53.23	26.04	0.001*
PLR	133.04	89.97	157.97	103.50	132.45	86.03	0.362
Systemic inflammatory index							
SIRI	0.63	0.58	1.13	1.24	0.59	0.52	<0.001*
SII	0.74	0.63	1.02	1.01	0.68	0.61	0.012*

* $p < 0.05$ indicates statistically significant related variables. CI: confidence interval, ABC: absolute basophil count, AEC: absolute eosinophil count, ANC: absolute neutrophil count, ALC: absolute lymphocyte count, AMC: absolute monocyte count, BLR: basophil to lymphocyte ratio, ELR: eosinophil to lymphocyte ratio, NLR: neutrophil to lymphocyte ratio, dNLR: derivative NLR, MLR: monocyte to lymphocyte ratio, MGLR: monocyte–granulocyte to lymphocyte ratio, BMR: basophil to monocyte ratio, EMR: eosinophil to monocyte ratio, NMR: neutrophil to monocyte ratio, LMR: lymphocyte to monocyte ratio, BPR: basophil to platelet ratio, EMR: eosinophil to platelet ratio, NMR: neutrophil to platelet ratio, LMR: lymphocyte to platelet ratio, MPR: monocyte to platelet ratio, PNR: platelet to neutrophil ratio, PLR: platelet to lymphocyte ratio, SIRI: systemic inflammatory response index, SII: systemic immune inflammation index.

Supplementary Table S2. ROC curve analysis of complete blood count-derived inflammatory biomarkers in infection after AIS.

Inflammatory biomarkers	Cut off	Sensitivity	Specificity	Youden Index	AUC	*p*-value	95% CI	
							Lower	Upper
Leukocyte count	9.55	0.708	0.741	0.45	0.744	<0.001*	0.622	0.866
Absolute leukocyte differential count								
ABC	0.15	0.042	0.993	0.03	0.470	0.635	0.348	0.591
AEC	0.28	0.292	0.863	0.16	0.500	1.000	0.359	0.641
ANC	6.88	0.667	0.799	0.47	0.754	<0.001*	0.634	0.875
ALC	1.29	0.958	0.144	0.10	0.448	0.419	0.327	0.569
AMC	0.27	0.750	0.633	0.38	0.710	0.001*	0.588	0.832
Lymphocyte ratios								
BLR	0.05	0.042	0.964	0.01	0.472	0.658	0.350	0.594
ELR	0.06	0.625	0.489	0.11	0.503	0.961	0.364	0.642
NLR	3.33	0.625	0.719	0.34	0.690	0.003*	0.582	0.799
dNLR	0.28	0.500	0.784	0.28	0.687	0.003*	0.575	0.799
MLR	0.21	0.750	0.424	0.17	0.585	0.182	0.469	0.702
MGLR	3.66	0.625	0.730	0.35	0.693	0.003*	0.585	0.801
Monocyte ratios								
BMR	0.17	0.083	0.917	0.008	0.469	0.633	0.349	0.590
EMR	0.84	0.375	0.670	0.04	0.458	0.515	0.325	0.592
NMR	10.45	0.750	0.460	0.21	0.621	0.058	0.507	0.735
LMR	3.19	0.750	0.250	0.00	0.418	0.198	0.300	0.535
Platelet ratios								
BPR	-	-	-	0.00	0.493	0.911	0.369	0.617
EPR	0.02	0.167	0.878	0.04	0.472	0.663	0.341	0.604
NPR	0.32	0.292	0.871	0.16	0.561	0.338	0.436	0.687
LPR	0.19	0.042	0.978	0.02	0.352	0.021*	0.232	0.472
MPR	0.06	0.042	0.986	0.03	0.437	0.323	0.319	0.554
Platelet count	327.50	0.417	0.719	0.14	0.534	0.592	0.402	0.667
Ratios of platelets to neutrophils and lymphocytes								
PNR	-	-	-	0.00	0.284	0.001*	0.166	0.401
PLR	154.93	0.542	0.66	0.20	0.558	0.362	0.430	0.687
Systemic inflammatory index								
SIRI	0.97	0.625	0.79	0.42	0.734	<0.001*	0.623	0.844
SII	0.61	0.875	0.44	0.31	0.662	0.012*	0.545	0.779

* $p < 0.05$ indicates statistically significant related variables. CI: confidence interval, ABC: absolute basophil count, AEC: absolute eosinophil count, ANC: absolute neutrophil count, ALC: absolute lymphocyte count, AMC: absolute monocyte count, BLR: basophil to lymphocyte ratio, ELR: eosinophil to lymphocyte ratio, NLR: neutrophil to lymphocyte ratio, dNLR: derivative NLR, MLR: monocyte to lymphocyte ratio, MGLR: monocyte-granulocyte to lymphocyte ratio, BMR: basophil to monocyte ratio, EMR: eosinophil to monocyte ratio, NMR: neutrophil to monocyte ratio, LMR: lymphocyte to monocyte ratio, BPR: basophil to platelet ratio, EMR: eosinophil to platelet ratio, NMR: neutrophil to platelet ratio, LMR: lymphocyte to platelet ratio, MPR: monocyte to platelet ratio, PNR: platelet to neutrophil ratio, PLR: platelet to lymphocyte ratio, SIRI: systemic inflammatory response index, SII: systemic immune inflammation index.
